# Nanoparticles of Quaternary Ammonium Polyethylenimine Derivatives for Application in Dental Materials

**DOI:** 10.3390/polym12112551

**Published:** 2020-10-30

**Authors:** Marta Chrószcz, Izabela Barszczewska-Rybarek

**Affiliations:** Department of Physical Chemistry and Technology of Polymers, Silesian University of Technology, 44-100 Gliwice, Poland; izabela.barszczewska-rybarek@polsl.pl

**Keywords:** dental composite restorative materials, antibacterial agents, quaternary ammonium polyethylenimine, nanoparticles

## Abstract

Various quaternary ammonium polyethylenimine (QA-PEI) derivatives have been synthesized in order to obtain nanoparticles. Due to their antibacterial activity and non-toxicity towards mammalian cells, the QA-PEI nanoparticles have been tested extensively regarding potential applications as biocidal additives in various dental composite materials. Their impact has been examined mostly for dimethacrylate-based restorative materials; however, dental cements, root canal pastes, and orthodontic adhesives have also been tested. Results of those studies showed that the addition of small quantities of QA-PEI nanoparticles, from 0.5 to 2 wt.%, led to efficient and long-lasting antibacterial effects. However, it was also discovered that the intensity of the biocidal activity strongly depended on several chemical factors, including the degree of crosslinking, length of alkyl telomeric chains, degree of N-alkylation, degree of N-methylation, counterion type, and pH. Importantly, the presence of QA-PEI nanoparticles in the studied dental composites did not negatively impact the degree of conversion in the composite matrix, nor its mechanical properties. In this review, we summarized these features and functions in order to present QA-PEI nanoparticles as modern and promising additives for dental materials that can impart unique antibacterial characteristics without deteriorating the products’ structures or mechanical properties.

## 1. Introduction

Dental composite materials are most commonly used for restorative applications. They are primarily based on dimethacrylate monomers, including bisphenol A glycerolate dimethacrylate (Bis-GMA), an ethoxylated Bis-GMA derivative (Bis-EMA), triethylene glycol dimethacrylate (TEGDMA), and/or urethane dimethacrylate (UDMA) [1,2]. In fact, almost 75% of dental composite materials contain Bis-GMA, and nearly 33% of them contain Bis-GMA and UDMA [2] because of their advantageous physico-chemical, mechanical, and esthetic properties. However, dental plaque accumulation on their surfaces is greater than that on the surfaces of teeth as well as other restorative materials, such as glass-ionomer cements and amalgams. This is due to higher surface roughness, higher surface free energy, and lack of antibacterial activity [3,4,5]. Bacteria that are collected onto the reconstruction surface can survive in marginal gaps that are formed between the reconstruction and adjacent tissue due to polymerization shrinkage [6,7,8]. Secondary caries can occur as a result of bacterial metabolic processes, which are believed to be the main cause of dental restoration failure [5,9,10]. Therefore, it is necessary to develop dental composite materials with antibacterial properties. The simplest way to accomplish this is by modifying their matrices with bioactive compounds that are physically dispersed within them. It is reported that particles of inorganic compounds, such as zinc oxide [5,11,12], titanium dioxide [13,14], calcium phosphate [15,16], gold [17,18,19,20], and silver [17,18,21,22], as well as organic compounds, such as antibiotics [23], chlorhexidine [5,24,25], furanone [5], ursolic acid [5], benzalkonium chloride [5], triclosan [5], chitosan [5], and methacryloyloxydodecyl pyridinium bromide (MDPB) [5], can be used for this purpose. The latter compound represents a dynamically developing group of antibacterial agents that are based on quaternary ammonium salts [26,27,28,29]. Recently, nanoparticles made up of crosslinked quaternary ammonium polyethylenimine derivatives (QA-PEI) have gained the attention of scientists.

**Figure 1:**
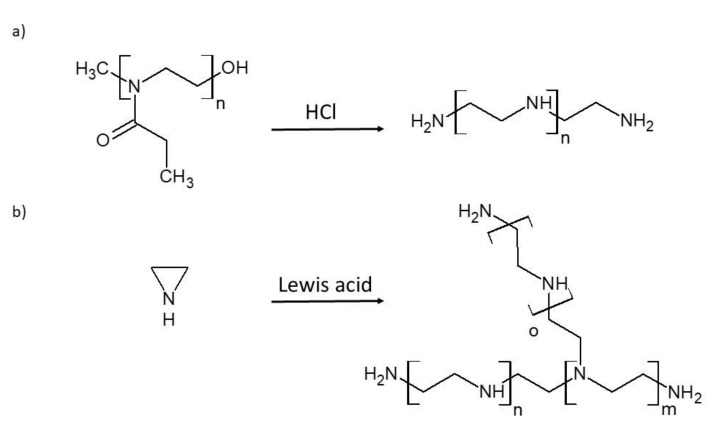
The synthesis of (**a**) linear polyethylenimine, (**b**) branched polyethylenimine. Adapted from [31].

Polyethylenimine (PEI), also known as polyaziridine, is a synthetic polymer whose repeating unit consists of linearly-arranged secondary amine groups and ethylene groups. PEI can exist in two structural architectures, linear or branched. Linear PEI (LPEI) is obtained via acidic hydrolysis of poly (2-ethyl-2-oxazoline), and its chemical structure contains only secondary amino groups (the primary amine end groups can be neglected due to their small number) [30]. Branched PEI (BPEI) is obtained through cationic polymerization of aziridine and contains primary, secondary, and tertiary amino groups (Figure 1) [31]. The main difference between LPEI and BPEI is that LPEI is a solid at room temperature (RT), whereas BPEI is a liquid at RT. The melting point of LPEI is about 73–75 °C and is soluble in chloroform, ethanol, methanol, and hot water at low pH. In addition, relative to BPEI, LPEI is characterized by stable chemical properties [32]. As a result of these favorable features, LPEI is more commonly used.

PEI has many potential applications due to its polycationic character and water solubility [33]. In the biomedical field, it can be used as an antiviral agent in DNA transfection because it is able to interact with negatively charged DNA and RNA [33,34,35,36]. It is used for separation and purification of biomacromolecules, enzyme immobilization, biosensor construction, and drug delivery [33,37,38,39,40,41,42]. PEI can additionally be applied as a heavy metal removal agent during water treatment [43,44,45,46,47], a dye removal agent in the paper and textile industry [48,49,50,51], in alkaline ion-exchange membranes [52,53,54,55], and in CO_2_ adsorbents [30,56,57,58,59]. The amino groups in PEI are highly reactive and, thus, can be easily modified in order to obtain quaternary ammonium polyethylenimine derivatives [60]. The quaternized forms of PEI (QA-PEI) exhibit antibacterial activity against Gram-positive and Gram-negative bacteria; therefore, they can be used as biocides [7,18,26,61,62]. They are also known to be effective at inactivating enveloped viruses, such as HBV, HCV, HIV, and influenza viruses [26,63]. It is worth noting that the U.S. Environmental Protection Agency currently recommends 216 products containing quaternary ammonium compounds for use to inactivate the SARS-CoV-2 (COVID-19) virus [64].

Due to the amphiphilic character of the QA-PEI repeating unit, consisting of the quaternary nitrogen atom substituted with one major hydrophobic chain, QA-PEI can effectively be applied as an antibacterial agent [25,30]. The mechanism of its action includes causing the disfunction of the lipid bilayer membranes constituting the bacterial cytoplasmic membrane and the outer-membrane of Gram-negative bacteria [25,65,66]. Initially, the positively charged ammonium group interacts with the negatively charged bacterial cell walls and disturbs its electrical balance by displacing divalent cations, such as Ca^2+^. Further, the hydrophobic substituent interdigitates into the hydrophobic membrane core to decrease membrane fluidity. Finally, the membrane loses many of its osmoregulatory and physiological functions. It is manifested as a cellular leakage of potassium ions (K^+^), protons, and other constituents of the cytoplasmic membrane, resulting in cell death [25,67,68,69,70,71,72,73,74,75,76]. In addition, the polycationic character of QA-PEI is responsible for its high antibacterial activity compared with low-molecular-weight antibacterial compounds [72,77]. All of these features make QA-PEI an attractive antibacterial agent for dental restorative composite materials.

Recent studies involving QA-PEI have focused on its crosslinked nanoparticulate forms. Such nanoparticles have been tested as antibacterial agents dispersed within the matrices of various dental materials used in restorative dentistry, orthodontics, prosthetics, endodontics, and oral implantology. The growing interest in dental nanomaterial applications for these nanoparticles is mainly due to their unique properties of nanoscale dimensions in combination with antibacterial activity [17,18,78]. Compared with micro- or macroparticles, nanoparticles are much more effective, and, therefore, they can be added in smaller quantities in order to achieve the desired function [17,18,78,79]. Additionally, by introducing QA-PEI nanoparticles into the dental materials, it is possible to achieve materials with antibacterial activity, while their original morphology and properties will be maintained.

This review comprehensively summarizes the results from several recent studies regarding (i) the synthesis of QA-PEI nanoparticles, (ii) their antibacterial activity as a function of chemical structure, (iii) the influence of incorporated QA-PEI nanoparticles on the antibacterial properties of various dental composite materials, and (iv) the impacts of incorporated QA-PEI nanoparticles on the macromolecular structure and mechanical properties of various dental composite materials.

## 2. Synthesis of QA-PEI Nanoparticles

QA-PEI nanoparticles are obtained from linear polyethylenimine. They were first synthesized by Beyth et al. [80] in 2006 via an N-alkylation method. This approach was later modified by Youdovin-Farber et al. [60] in 2010, who employed a reductive amination reaction.

**Figure 2:**
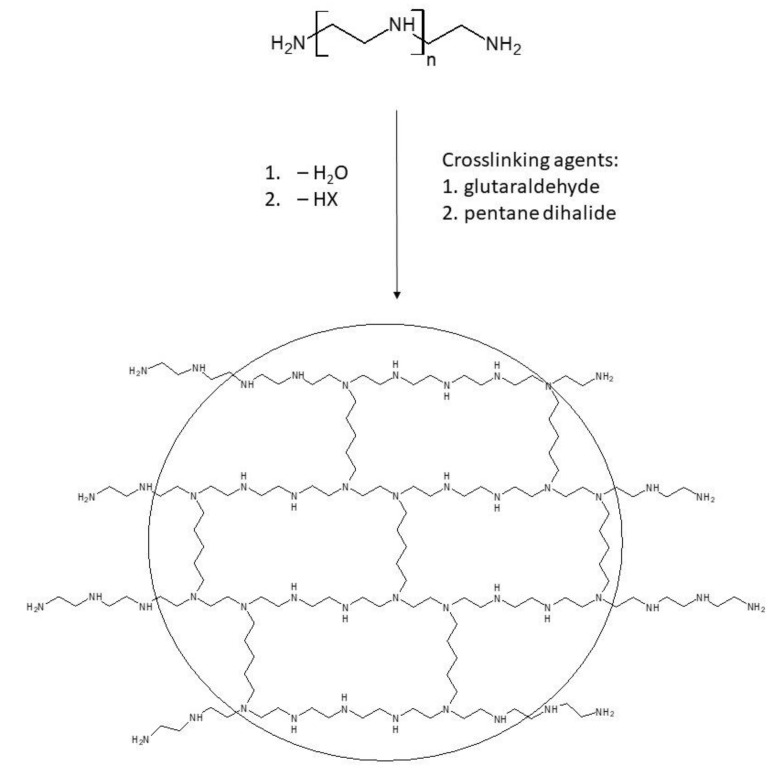
Formation of crosslinked polyethylenimine (PEI) nanoparticles. Adapted from [60].

Both processes include the following key steps:

1. Crosslinking: The goal of this process is to form PEI nanoparticles that consist of a crosslinked core with a large number of free-standing side chains. Due to the covalent bonding of the side chains, they represent an integral part of the core and cannot be dissociated [81]. The overall structure of a PEI nanoparticle contains primary, secondary, and tertiary amino groups. Primary amino groups are located at the ends of side chains, secondary amino groups are located within the core and the side chains, and tertiary amino groups are only located in the core. The difference between the two crosslinking methods is that, in the reductive amination process, glutaraldehyde is used as a crosslinking agent, whereas in the N-alkylation process, crosslinking occurs using an alkyl dihalide, usually 1,5-dibromopentane (Figure 2) [60]. 1,4-dibromobutane and 1,6-dibromohexane can also be used as suitable crosslinking agents [82].

It is recommended to use a crosslinker in amounts from 1 to 20 mol.% [82]. The crosslink density influences the physico-chemical properties of QA-PEI nanoparticles. High crosslink density can cause an excessive stiffening of QA-PEI nanoparticles, decrease in their positive charge, and result in dimensions that are too large. This was demonstrated by Farah et al. [83], who performed crosslinking of PEI with the use of 1,5-diiodopentane in the amounts of 4, 8, and 12 mol.%. An increase in the crosslink density resulted in the PEI stiffening, which was manifested by the increase in glass temperature (T_g_). Its value increased from 148 to 161 °C. Moreover, an increase in the crosslink density caused a decrease in the number of positively charged QA groups per surface area, and an increase in dimensions of nanoparticles (nanoparticles of 30 µm in diameter were generated when 12% of crosslinker was used). The crosslinker content did not influence the hydrophobicity of QA-PEI nanoparticles.

**Figure 3:**
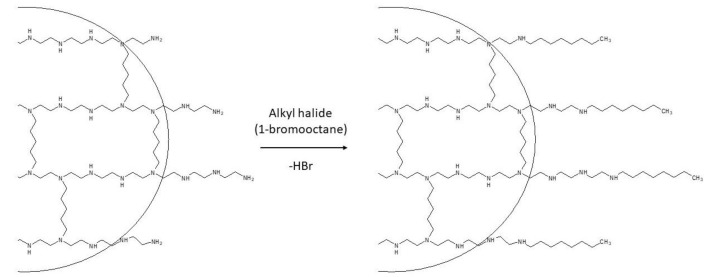
Telomerization in the N-alkylation method with the use of 1-bromooctane. Adapted from [60].

**Figure 4:**
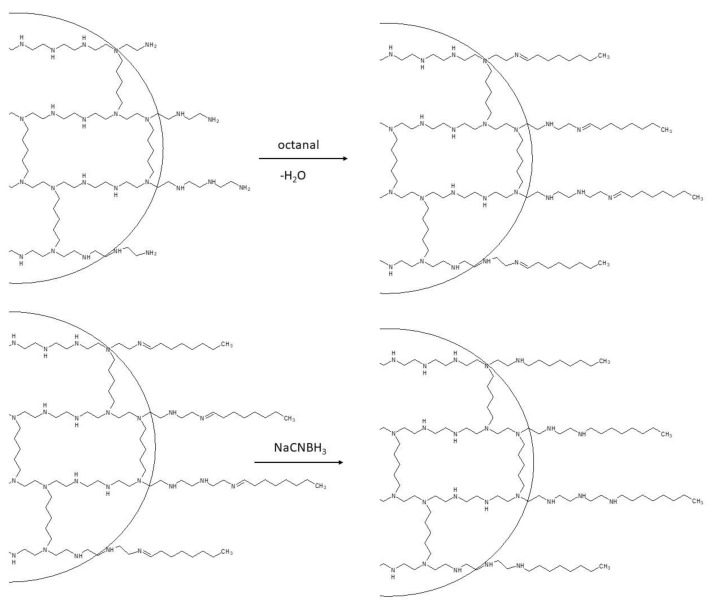
Telomerization in the reductive amination method with the use of octanal. Adapted from [60].

2. Telomerization: This step aims to extend the side chains by adding long linear alkyl chains, which are crucial for defining the hydrophobic character of the nanoparticles. In fact, hydrophobicity governs the interactions of QA-PEI nanoparticles with lipids located in bacterial cell walls. The telomerization process involves the transformation of primary amino groups into secondary amino groups. Alkyl bromide is often used for this purpose in the *N*-alkylation method (Figure 3), whereas octanal is used in the reductive amination method (Figure 4).

The reductive amination method requires an additional step, which involves the reduction of the double bonds that are formed during the reaction of the primary amino groups with octanal. Sodium cyanoborohydride (NaCNBH_3_) is used in this reaction as a reducing agent (Figure 4) [60,82].

Octyl bromide is the most often used N-alkylation agent [60,84,85]. Butyl bromide [85], hexyl bromide [85,86], decyl bromide [85,86], dodecyl bromide [84], hexadecyl bromide [84,85], and α-bromotoluene [86] may also be used as suitable N-alkylation agents.

It is recommended to determine the primary amine content after this stage, which could be performed utilizing the trinitrobenzene sulfonic acid (TNBS) method [87].

The important aspect of this stage is the neutralization of hydrogen bromide (HBr), which is formed in the *N*-alkylation method. Typically, sodium bicarbonate (NaHCO_3_) is used for this purpose [60]. Zaltsman et al. [88] tested the influence of variations in the *N*-alkylation stage on the manufacturing process of QA-PEI nanoparticles. Those variations included (i) the controlled neutralization of hydrogen iodide with a minimal amount of NaHCO_3_; (ii) in the case of excess NaHCO_3_, the neutralization of the base with strong hydrochloric and phosphoric acids; (iii) treatment with surfactants *N*-lauroylsarcosine or glycerol monostearate. The application of glycerol monostearate resulted in the formation of fibrous QA-PEI derivatives; therefore, this could not be considered further. The efficiency of all other methods was assessed by testing the total bacterial growth inhibition on the surface of the dimethacrylate-based dental restorative material (Filtek Supreme XT) modified with QA-PEI nanoparticles. The highest antibacterial activity was achieved for the standard neutralization procedure and those involving phosphoric acid and N-lauroylsarcosine surfactant. The total bacterial growth inhibition for them was achieved with a content of 0.5 wt.% QA-PEI nanoparticles. A greater amount of QA-PEI nanoparticles, corresponding to 1 wt.%, was required to achieve total bacterial growth inhibition with QA-PEI nanoparticles obtained via controlled neutralization and the application of hydrochloric acid. The findings lead to the conclusion that the standard method of the HBr neutralization can be recommended. Tested modifications are of no particularly great benefit as they require the application of additional reagents, which is not accompanied by an increase in the antibacterial activity.

**Figure 5:**
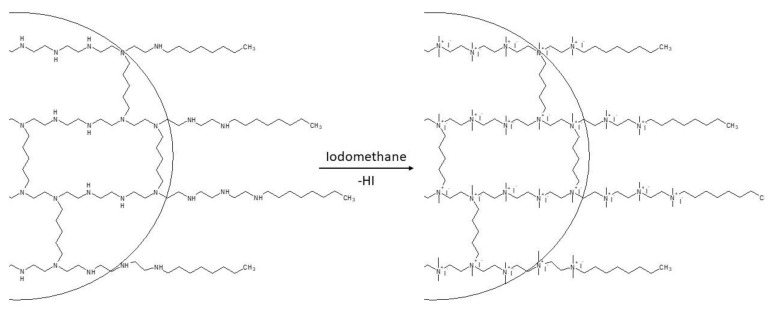
The quaternization step that occurs in both methods. Adapted from [60].

3. Quaternization: This process results in the generation of quaternary ammonium groups. Specifically, the procedure targets secondary and tertiary amino groups that can react with an alkyl halide, which functions as the quaternization agent. Since iodomethane is typically used for this purpose, this step is also called *N*-methylation (Figure 5) [60]. The quaternization of amine groups is responsible for instilling the QA-PEI nanoparticles with their antibacterial properties. This stage is also crucial for determining the intensity of their antibacterial activity [60,85,86].

The synthesis of QA-PEI nanoparticles is typically carried out in polar, anhydrous solvents. Absolute ethyl alcohol (anhydrous) is the most commonly used because it is highly polar and can prevent precipitation of nanoparticles, which often occurs in the presence of moisture.

The original *N*-alkylation method of QA-PEI nanoparticle synthesis has been proven to be the easiest and least time-consuming; therefore, this approach is more commonly used [80,83,84,85,86,88,89,90,91,92,93,94,95,96,97,98] than the reductive amination [60].

QA-PEI nanoparticles prepared via the reductive amination and *N*-alkylation methods can differ in diameter [60,82,83]. According to its U.S. patent, QA-PEI nanoparticles in sizes between 30 and 150 nm are the most preferable. However, nanoparticles smaller than 1000 nm are also acceptable [82]. The length and content of the *N*-alkylation agent influence the QA-PEI nanoparticles’ size. For example, it was found that complete *N*-alkylation using octyl bromide resulted in the formation of QA-PEI nanoparticles with the apparent particle size of 24 and 40 nm, respectively [60]. A decrease in the octyl halide content resulted in an increase in the QA-PEI nanoparticles’ dimensions. For example, QA-PEI nanoparticles alkylated with 25 mol.% of octyl halide with respect to PEI units were characterized by a particle size from 160 to 190 nm [83]. The increase in the *N*-alkylation agent length also caused an increase in the nanoparticle size [85]. An increase in the *N*-alkyl substituent length from 4 to 10 carbon atoms resulted in an increase in the QA-PEI nanoparticles’ diameter from 200 to 500 nm.

These findings lead to the conclusion that QA-PEI nanoparticles can be effectively synthesized via the *N*-alkylation method, show inhibition of bacterial growth, and, therefore, could be used as antibacterial additives for biomedical devices [60]. However, the recognition that the antibacterial activity of QA-PEI nanoparticles is governed by particular elements of their chemical structure has initiated a research path exploring the relationships between the chemical structure of QA-PEI nanoparticles and their biocidal properties.

## 3. Antibacterial Properties of QA-PEI Nanoparticles

Many studies have shown that QA-PEI nanoparticles exhibit antibacterial activity against various strains of bacteria. Their biocidal properties have been examined against Gram-positive bacteria (*Actinomyces viscosus* [92,93], *Enterococcus faecalis* [83,88,90,93], *Lactobacillus casei* [93], *Staphylococcus aureus* [60,84,90], *Staphylococcus epidermidis* [90], *Streptococcus mutans* [80,85]), as well as Gram-negative bacteria (*Escherichia coli* [36,90] and *Pseudomonas aeruginosa* [84,86,90]).

The antibacterial properties of QA-PEI nanoparticles are significantly affected by the reagent types and quantities used in their synthesis. The choice of reagents ultimately governs various structural parameters of QA-PEI nanoparticles, including the degree of crosslinking, telomere length, degree of telomerization, degree of quaternization, and counterion type. The antibacterial properties of QA-PEI nanoparticles can also be affected by environmental factors, such as pH or the presence of oxidizing agents.

QA-PEI nanoparticles are usually tested for antimicrobial activity (*A*), which can be calculated according to the following equation:

(1) $$A=logB_{0}-log\frac{\sum_{i=1}^{n}B_{i}}{n}$$

where *logB*_0_ is the initial suspension concentration (in CFU/mL; CFU—colony forming units), *B_i_* is the suspension concentration after incubating with QA-PEI samples (in CFU/mL), and *n* represents the number of replicated tests.

### 3.1. Crosslink Density

Crosslinking is the first step in QA-PEI nanoparticle synthesis, and it is responsible for creating the nanoparticulate form, causing insolubility [90], and generating “non-leaching” antimicrobial polymers [80,83].

The crosslinking agent type and crosslink density influence the antibacterial activity of QA-PEI nanoparticles.

The effect of the crosslinking agent on the antimicrobial activity of QA-PEI nanoparticles against Gram-positive (*S. aureus*) and Gram-negative (*P. aeruginosa*) bacteria was shown in the work of Nuzhdina et al. (Table 1) [84]. The following crosslinking agents were used for this purpose: ethylene glycol bis(chloroacetate), diethylene glycol bis(chloroacetate), triethylene glycol bis(chloroacetate), polyethylene glycol bis(chloroacetate), 1,5-dibromopentane, and glutaraldehyde. All of them were applied at the constant mole ratio of PEI monomer units to crosslinking agent, which was 1:0.04.

As can be seen from Table 1, the QA-PEI nanoparticles showed antibacterial activity against *S. aureus*. Its value depended on the bacteria class and crosslinker type.

It is not surprising that tested QA-PEI nanoparticles demonstrate low antibacterial activity against *P. aeruginosa* [84]. This is a highly pathogenic strain of Gram-negative bacteria. This bacteria is characterized by the bilayer structure of its cell membrane, which is built of rigid lipopolysaccharides and makes them highly resistant to many antimicrobial agents [99,100,101].

The antibacterial activity of QA-PEI nanoparticles also depends on the hydrophilic character of the crosslinker [84]. For example, the glycol-based crosslinkers, which are more hydrophilic than commonly used 1,5-dibromopentane and glutaraldehyde, displays more efficient antibacterial activity. Additionally, an increase in the antibacterial activity is observed with the increase in the crosslinker oligooxyethylene chain length. It is well known that increasing the length of the oligooxyethylene chain results in an increase in its elasticity and the same occurs with the increase in polymer network elasticity [102]. Therefore, it can be assumed that the antibacterial activity of the QA-PEI nanoparticles depends on the elasticity of the polymer network, constituting their cores. The greater the core elasticity, the greater the antibacterial activity.

The antibacterial activity of QA-PEI nanoparticles is also influenced by the crosslink density. The higher the concentration of the crosslinking agent, the lower the antibacterial activity. This can be linked to the increasing nanoparticle core stiffness (an increase in T_g_ is observed with an increase in crosslink density [83,102]). The effect of the crosslink density on the antibacterial activity of QA-PEI nanoparticles was shown by Nuzhdina et al. [84]. They observed that an increase in the content of ethylene glycol bis(chloroacetate), used as a crosslinker in a molar ratio ranging from 0.02 to 0.10 with respect to PEI monomer units, caused a decrease in the antibacterial activity against *S. aureus* as well as *P. aeruginosa* (Table 2).

Youdovin-Farber et al. [85] confirmed that antibacterial activity decreased as the crosslink density increased and additionally revealed that insufficient crosslink density could cause a decrease in the antibacterial activity of QA-PEI nanoparticles. The crosslinking was performed with 1,5-dibromopentane at concentrations of 0.01, 0.04, and 0.2 mole fraction with respect to PEI monomer units. The antibacterial activity of the resulting QA-PEI nanoparticles was evaluated by determining the bacterial growth (%) of *S. mutans* on a restorative composite resin enriched with 1 wt.% QA-PEI nanoparticles. The QA-PEI nanoparticles of moderate crosslink density, corresponding to 4 mol.% of the crosslinker, displayed the most effective antibacterial action (bacterial growth on the modified composite corresponded to 0.2%). In contrast, QA-PEI nanoparticles synthesized using 1 and 2 mol.% of the crosslinker inhibited bacterial growth 330 and 75 times less efficiently, respectively (i.e., bacterial growth on the modified composite corresponded to 66% and 15%, respectively). The weakest bacterial growth inhibition, exhibited by nanoparticles of the lowest crosslink density, could be attributed to the insufficient crosslink density, which led to the separation of the polymer chains. In the case of the QA-PEI nanoparticles with the highest crosslink density, the nanoparticles’ core was probably too stiff, so their interaction with the bacterial membrane was reduced, and the antibacterial activity of the sample decreased.

To conclude, it is clear that the crosslink density in the core of QA-PEI nanoparticles strongly influences their antibacterial activity and must be carefully examined to design nanoparticles with optimal performance. A crosslink density that is either too large or too small may have detrimental effects.

### 3.2. Length of N-alkyl Telomers

QA-PEI nanoparticles contain long side chains with alkyl telomeres at the ends [60,80,82]. These telomeres originate from *N*-alkylation (using alkyl halides) of the side chains that protrude from the crosslinked PEI core. Alkyl bromides are used in most cases because of their high reactivity in nucleophilic substitution reactions, which is related to their acidity (more reactive than alkyl chlorides and alkyl fluorides, but less reactive than alkyl iodides). During the nucleophilic substitution, the nucleophile (in this case, an amine) displaces the leaving group of an acyl derivative (in this case, an alkyl halide). As the nucleophile takes the place of the leaving group, the reactivity of the acyl derivative depends on the propensity of the halogen group to leave. Weak bases (anions of strong acids) are better leaving groups than strong bases. Since hydroiodic and hydrobromic acids are much stronger acids than hydrofluoric or hydrochloric acids, the iodide and bromide anions represent better leaving groups than fluoride and chloride anions; therefore, they have higher reactivity in nucleophilic substitution reactions [103,104].

PEI is hydrophilic, and alkyl chains are hydrophobic, so the *N*-alkylation process decreases the hydrophilicity of QA-PEI nanoparticles. Longer alkyl bromide chains and greater quantities of introduced alkyl bromide both lead to stronger hydrophobic character in the resulting QA-PEI nanoparticles [81]. Hydrophobic character is crucial for the antibacterial efficiency of QA-PEI nanoparticles because after diffusing through the bacterial cell wall, antibacterial agents with hydrophobic moieties can bind to the cytoplasmic membrane [25,72]. For this reason, the relationship between the length of alkyl bromides used and the resulting antibacterial properties of QA-PEI nanoparticles requires detailed understanding.

The results of several studies show that the antibacterial activity of QA-PEI nanoparticles is strongly affected by the *N*-alkyl chain length. It increases with the increasing length of the alkyl telomere from four-carbon chains up to eight-carbon chains. However, increasing the alkyl chain length any further reduces the antibacterial activity of the QA-PEI nanoparticles.

**Figure 6:**
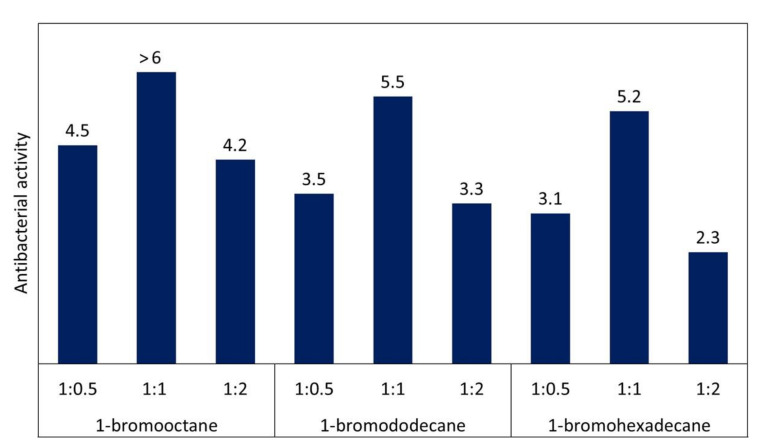
Antibacterial activity of QA-PEI nanoparticles alkylated with different *N*-alkylation agents and with various degrees of *N*-alkylation. Data from [84].

Nuzhdina et al. [84] showed that QA-PEI nanoparticles with octyl and dodecyl telomeres exhibited significantly higher antibacterial activity against *S. aureus* relative to nanoparticles containing hexadecyl telomers. QA-PEI nanoparticles obtained with 1-bromooctane and 1-bromododecane attained CFU/mL values on the order of 1 × 10^6^, whereas those obtained with 1-bromohexadecane had 50% lower CFU/mL values (5 × 10^5^). In the same study, the antibacterial activity of QA-PEI nanoparticles was also calculated using Equation (1). The data presented in Figure 6 shows that the use of 1-bromooctane as the *N*-alkylation agent resulted in QA-PEI nanoparticles with the highest antibacterial activity.

**Figure 7:**
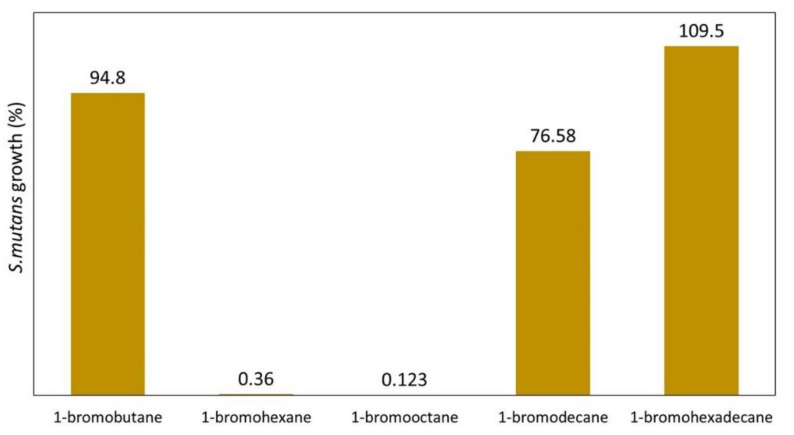
*S. mutans* growth (%) on the surface of a commercial dental composite enriched with 1 wt.% QA-PEI nanoparticles, which were telomerized using alkyl bromides of various lengths. Data from [85].

Youdovin-Farber et al. [85] showed the detailed relationship between the antibacterial efficiency of QA-PEI nanoparticles and *N*-alkyl chain length. The presence of the 4-carbon chain resulted in very low antibacterial efficiency. The extension of the *N*-alkyl substituent, having 6 and 8-carbon chains, resulted in a radical increase in the antibacterial efficiency, and it reached a maximum for the 8-carbon chain. Further increase in the *N*-alkyl chain length resulted in a radical decrease in the antibacterial efficiency of QA-PEI nanoparticles. The QA-PEI nanoparticles obtained using 1-bromooctane inhibited the growth of *S. mutans* almost 800 times better than those obtained using 1-bromobutane, and almost 900 times better than those obtained using 1-bromohexadecane (Figure 7).

**Figure 8:**
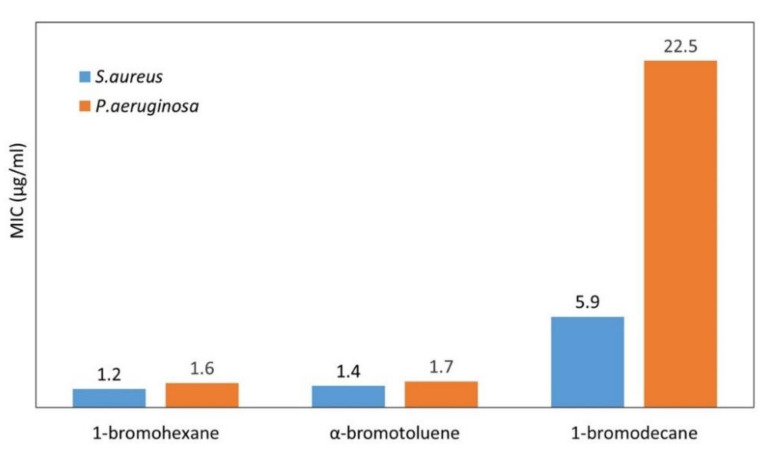
The minimum inhibitory concentration (MIC) (µg/mL) values of fully quaternized linear QA-PEIs obtained using various bromides and tested against *S. mutans* and *P. aeruginosa*. Data from [86].

By testing the microbiological activity of the linear QA-PEIs, Yew et al. [86] confirmed that the longer the *N*-alkyl chain, the weaker the antibacterial action. The QA-PEI modified with 1-bromohexane exhibited greater antibacterial activity than that modified with 1-bromodecane. The minimum inhibitory concentration (MIC) values of QA-PEI with decyl telomeres was almost five times higher than that of QA-PEI with hexyl telomeres in the tests against *S. aureus* and about 14 times higher in the tests against *P. aeruginosa* (Figure 8). Yew et al. [86] also showed that the utilization of aryl halide (α-bromotoluene), instead of the aliphatic *N*-alkylation agent, resulted in high antibacterial efficiency of the QA-PEI, which was comparable to that of the QA-PEI modified with 1-bromohexane.

From the above, it is seen that octyl substituent results in the highest antibacterial activity of QA-PEI nanoparticles. *N*-alkyl substituents, which have less or more than eight carbon atoms, decrease antibacterial activity. The finding is in agreement with the literature data on the general antibacterial activity of quaternary ammonium compounds [25], which says that compounds with *N*-alkyl chain lengths of less than 4 and more than 18 carbon atoms are virtually inactive.

The poor antibacterial activity of QA-PEI nanoparticles obtained with bromobutane is likely due to their insufficient hydrophobicity, in addition to the fact that short alkyl chains are not able to effectively interact with bacterial cells’ lipids [85].

QA-PEI nanoparticles with excessively long telomeres have increased hydrophobic character and, therefore, they also exhibit reduced antibacterial activity. Long, hydrophobic telomeres impart an amphiphilic character, which results in an increased tendency to form micelles. Such micellization decreases the ability of QA-PEI nanoparticles to lyse bacterial cells, thereby reducing their antibacterial activity [86]. Alternatively, steric effects caused by the long alkyl chains may also influence their activity. If there are more than eight carbon atoms in the telomers, the positively charged quaternary nitrogen atoms might be screened, thus reducing their interactions with negatively-charged bacterial cell walls [84]. Finally, longer telomeres are capable of adhering to one another via hydrophobic intermolecular interactions, which may also hinder the access of the nitrogen atoms [85,86].

### 3.3. Degree of N-Alkylation

The antibacterial properties of QA-PEI nanoparticles strongly depend on the degree of *N*-alkylation, also called the degree of telomerization, which is determined by the molar ratio of the telomerization agent to PEI units. A larger molar fraction of the telomerization agent leads to a greater number of primary amino groups that are converted to secondary amino groups, which is the metric of the degree of *N*-alkylation. A higher degree of *N*-alkylation indicates an increase in the hydrophobic character of the QA-PEI nanoparticles, which enables favorable interactions with the lipids of bacterial cell walls [25]. Therefore, the higher the degree of *N*-alkylation, the higher the antibacterial activity of QA-PEI nanoparticles [60,84,85]. A low degree of *N*-alkylation decreases the hydrophobic character of QA-PEI nanoparticles, so their ability to bind with the cytoplasmic membrane after diffusing through the bacterial cell walls is lower, thus decreasing their antibacterial efficiency [25]. Therefore, incomplete *N*-alkylation usually leads to insufficient antibacterial activity of QA-PEI nanoparticles [60,84,85]. On the other hand, the application of excessive amounts of *N*-alkylation agent has the same negative effect [84].

**Figure 9:**
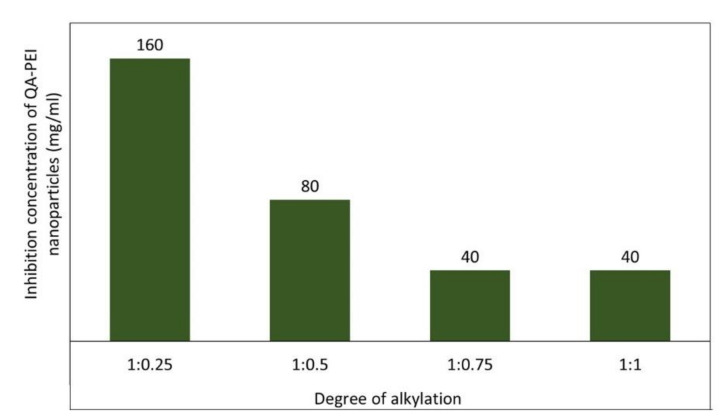
Concentration of QA-PEI nanoparticles (mg/mL) with varying degrees of telomerization required for complete inhibition of *S. aureus* growth. Data from [60].

Youdovin-Farber et al. [60] showed that the antibacterial efficiency of QA-PEI nanoparticles increased with an increasing degree of *N*-alkylation by using octanal as the *N*-alkylation agent in molar fractions with respect to PEI units ranging from 0.25 to 1 (Figure 9). The QA-PEI nanoparticles obtained with 75 and 100 mol.% of octanal inhibited bacterial growth at concentrations four times lower than nanoparticles obtained with 25 mol.% of octanal. This result indicates that a 75 mol.% content of the *N*-octyl substituent relative to PEI unit is sufficient to achieve a satisfactory antibacterial effect against *S. aureus*, and that increasing this mole fraction any further adds no benefit.

Youdovin-Farber et al. [85] showed that incomplete telomerization negatively affected the antibacterial efficiency of QA-PEI nanoparticles. The antibacterial efficiency was tested against *S. mutans* by the modification of a restorative composite with 1 wt.% QA-PEI nanoparticles and determination of bacterial growth (%) onto a composite surface. The completely-telomerized QA-PEI nanoparticles (1:1 mol/mol PEI units/bromooctane) were almost six times more efficient in inhibiting the growth of *S. mutans* compared to those telomerized at 25%. The 1:1 mol/mol PEI units/bromooctane mole ratio resulted in 0.123% bacterial growth on the investigated surfaces, whereas the mole ratio 1:0.25 resulted in 0.7% bacterial growth.

Nuzhdina et al. [84] also showed that a low degree of telomerization led to insufficient antibacterial activity of the QA-PEI nanoparticles. Additionally, they showed that the utilization of excessive amounts of the *N*-alkylation agent resulted in decreased antibacterial activity of QA-PEI nanoparticles. In their study, QA-PEI nanoparticles were telomerized using three different alkyl bromides (having 8, 12, or 16-carbon chains) added in various mole ratios of PEI units to alkyl bromide (1:0.5, 1:1, or 1:2). The greatest antibacterial activity of QA-PEI nanoparticles (against *S. aureus*) was achieved with a stoichiometric amount of alkyl bromide (1:1 mol/mol) in each series (Figure 6). Further, within the most bioactive series (employing bromooctane), the use of a 1:1 mol/mol ratio of PEI units/bromooctane increased the antibacterial efficiency by 33% and 43% relative to the 1:0.5 and 1:2 mol/mol ratios, respectively. In the least bioactive series (employing bromohexadecane), complete telomerization with a 1:1 mol/mol ratio of PEI units/bromohexadecane induced the most dramatic increase in antibacterial efficiency. Its value was 68% and 126% greater than the 1:0.5 and 1:2 mol/mol ratios of PEI units/bromohexadecane, respectively.

### 3.4. Degree of Quaternization

Quaternization is the final step in the manufacturing process of QA-PEI nanoparticles. This procedure involves the reaction between the tertiary nitrogen atom resulting from *N*-alkylation and methyl iodide to form quaternary ammonium salt. When the permanently-charged quaternary ammonium cation is formed, the PEI derivative gains its antimicrobial activity, the intensity of which depends on the amount of methyl iodide used in the reaction. In general, a higher mole fraction of methyl iodide leads to a greater degree of cationization (a greater number of quaternary ammonium cations) and a greater ability of the QA-PEI derivatives to interact with negatively-charged bacterial cell surfaces [36].

**Figure 10:**
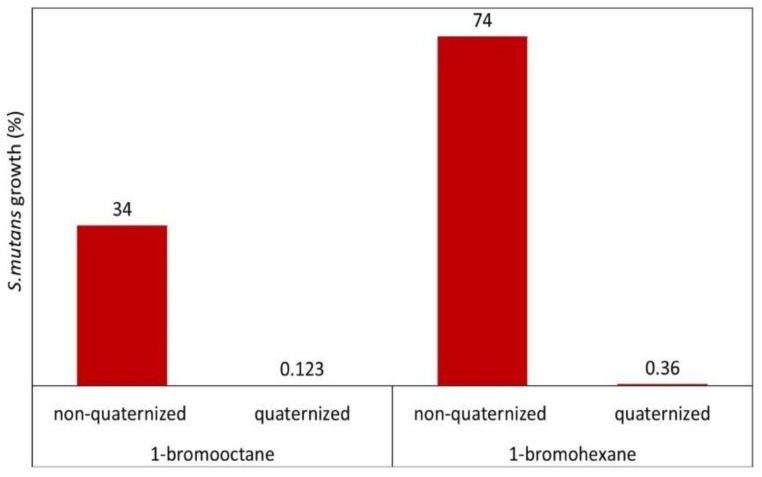
*S. mutans* growth (%) on the surface of a restorative composite resin containing 1 wt.% quaternized and non-quaternized QA-PEI nanoparticles. Data from [85].

The *N*-methylation has a significant influence on the QA-PEI nanoparticles’ antibacterial properties. The methylated form of QA-PEI nanoparticles inhibit bacterial growth several times more efficiently than their non-methylated counterparts. The study of Youdovin-Farber et al. [85] revealed this difference. They tested the antibacterial activity of a restorative composite enriched with 1 wt.% of methylated or non-methylated QA-PEI nanoparticles, having octyl and hexyl telomers, against *S. mutans*. The methylated forms of QA-PEI nanoparticles inhibited bacterial growth (%) almost 300 times and 200 times more efficiently than their non-methylated counterparts, respectively (Figure 10).

The results of another study by Youdovin-Farber et al. [60] revealed that QA-PEI nanoparticles gain antibacterial activity at a particular degree of quaternization. An insufficient methylation degree obstructed the antibacterial activity of QA-PEI nanoparticles. In the study, QA-PEI nanoparticles with octyl telomeres were subjected to quaternization using methyl iodide. The mole ratio of the PEI monomer units to methyl iodide ranged from 1:1 to 1:3. QA-PEI nanoparticles obtained using either 1:1 or 1:2 mole ratios did not show antibacterial activity against *S. aureus* at concentrations up to 80 µg/mL, suggesting an insufficient degree of *N*-methylation. However, the QA-PEI nanoparticles obtained using a 1:3 mol/mol ratio (monomer units of PEI/methyl iodide) completely inhibited bacterial growth at a concentration of 80 µg/mL.

The studies on linear QA-PEI suggest that the antibacterial activity of QA-PEI nanoparticles can result from the combined effect of the quaternization degree, *N*-alkylation agent, and bacteria strain. Randomly chosen reactants can strongly limit antibacterial activity. On the other hand, a carefully selected combination of reactants supported with microbiological tests can trigger antibacterial activity against a particular bacteria strain.

**Figure 11:**
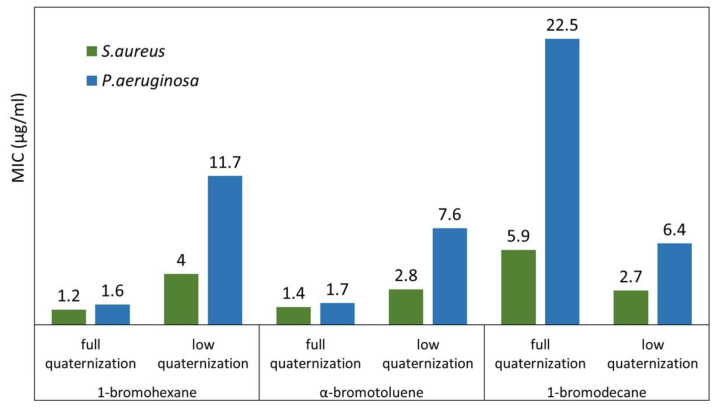
MIC (µg/mL) values measured for linear QA-PEI, distinguished by their quaternization degree and *N*-alkyl substituents, in tests against *S. aureus* and *P. aeruginosa*. Data from [86].

Yew et al. [86] showed the combined influence of the degree of quaternization and telomere type on the antibacterial efficiency of the linear QA-PEI against *S. aureus* and *P. aeruginosa*. The completely quaternized forms of QA-PEI nanoparticles modified with 1-bromohexane and α-bromotoluene had higher antibacterial activity than their partially quaternized counterparts. When 1-bromodecane was used, a reverse effect was observed (Figure 11). Fully quaternized QA-PEI, *N*-alkylated with 1-bromohexane, produced 70% and 86% lower MIC values against *S. aureus* and *P. aeruginosa* than their incompletely quaternized counterparts, respectively. A similar trend was observed for the QA-PEI *N*-alkylated with α-bromotoluene. As the degree of quaternization increased, the MIC values decreased by 50% and 78%, respectively. The MIC values that emerged with QA-PEI *N*-alkylated with 1-bromodecane, on the contrary, increased respectively by 119% and 252%, due to complete quaternization. These results revealed the influence of bacteria class on the QA-PEI antibacterial efficiency. *P. aeruginosa* exhibited higher resistance to QA-PEI compared to *S. aureus*. Differences in the cell wall structure between Gram-positive and Gram-negative bacteria are responsible for this effect. Gram-positive bacteria, represented here by *S. aureus*, have a loosely packed polyglycane cell wall, which facilitates its deterioration due to the interaction with QA-PEI. Gram-negative bacteria, represented here by *P. aeruginosa*, have an additional membrane composed of a phospholipid bilayer, which acts as a barrier against QA-PEI [65,66,105].

The influence of the quaternization degree on the antibacterial activity of linear QA-PEI was confirmed by Gao et al. [36] in tests against *E. coli*. Propylene oxide was used as the *N*-alkylation agent and benzyl chloride as the quaternization agent. The increase in the quaternization degree resulted in a decrease in the QA-PEI concentration required to kill the bacteria. They reported that QA-PEI with a quaternization degree of 54.9%, at a concentration of 15 µg/mL, killed almost all *E. coli* bacteria after four minutes, whereas QA-PEI with a quaternization degree of 40.8%, at the same concentration, killed only about 80% of bacteria over the same time.

### 3.5. Effect of Counterion

As mentioned before, QA-PEI derivatives inactivate bacteria by disrupting the cellular membrane due to electrostatic interaction with negatively charged bacteria and the replacement of Ca^2+^ and/or Mg^2+^ by biocidal cations. In this regard, the identity of the counter anion could play a role in the antimicrobial performance of polymeric quaternary ammonium salts. However, many studies on polymeric quaternary ammonium salts show that the counter anions, such as chloride, bromide, iodide, hydroxide, and phosphate, play a minor role in their biocidal performance [105]. No difference in the antimicrobial behavior is shown for QA-PEI nanoparticles.

The effect of the counterion type on the antibacterial activity of QA-PEI nanoparticles was demonstrated by Youdovin-Farber et al. [60], who partially replaced the original iodide anions with nitrate or acetate counterions. The antibacterial activity of the QA-PEI nanoparticles was then determined against *S. aureus*. The concentration of QA-PEI nanoparticles required to inhibit bacterial growth was 40 µg/mL for QA-PEI nanoparticles with nitrate and acetate anions and 80 µg/mL for QA-PEI nanoparticles with iodide counter anions. These results led to the conclusion that the anion type did not cause significant differences in the bacterial growth inhibition exhibited by QA-PEI nanoparticles, which was consistent with the results achieved in the study regarding linear QA-PEI derivatives.

### 3.6. Relationship Between the pH and Antibacterial Properties of QA-PEI Nanoparticles

In addition to the chemical structure, the pH can be recognized as a physical factor affecting the antibacterial properties of QA-PEI nanoparticles.

QA-PEI has minimal antibacterial activity at the pH value corresponding to the isoelectric point of the bacteria, which represents the pH where bacterial cell surfaces are not charged, thus disallowing the interaction between these surfaces and the positively-charged nanoparticles [36].

Dental materials operate in the oral cavity in a saliva environment. In the oral cavity, the pH is maintained near neutrality by saliva and ranges from 6.7 to 7.3 [106]. Therefore, it is important to study the antibacterial activity of QA-PEI nanoparticles at various pH, in particular within the range mentioned above.

The relationship between the antibacterial activity of QA-PEI nanoparticles against salivary bacteria at various pH still requires testing. Gao et al. [36] tested this relationship for the linear QA-PEI against *E. coli*. The isoelectric point of the protein of *E. coli* cells was observed at pH 4.5. QA-PEI emerged with negligible antibacterial efficiency at this pH. As the pH increased above 4.5, the antibacterial activity of QA-PEI increased. The antibacterial activity maximum was observed at pH 6 and higher due to the strengthening of the electrostatic interactions between the positively-charged QA-PEI and the negatively-charged bacterial cell surfaces.

### 3.7. Effect of Oxidizing Environment

An interesting issue related to the antibacterial activity of QA-PEI nanoparticles is the influence of aggressive environments. Farah et al. [83] studied the effect of different oxidizing agents, including hydroxide peroxide, oxygen, and air, on the chemical stability of QA-PEI nanoparticles and their antibacterial activity against *E. faecalis*. The QA-PEI nanoparticles exhibited high chemical stability as no changes in the chemical structure or effective positive charge were observed after exposure for one week to different oxidizers. The antibacterial activity of aged QA-PEI nanoparticles was assessed by testing the antibacterial action of the commercial dental composite enriched with QA-PEI nanoparticles in the amounts of 0.5, 1, and 2 wt.%. The exposure of QA-PEI nanoparticles to hydrogen peroxide for one week resulted in the highest antibacterial activity of modified composites and ultimately resulted in total bacterial growth inhibition at a QA-PEI nanoparticle concentration of 1 wt.%. The total inhibition of bacterial growth for composites modified with the QA-PEI nanoparticles exposed to O_2_ for one week was achieved at a concentration of 2 wt.%. The samples that were exposed to air induced total bacterial growth inhibition along with a QA-PEI nanoparticle concentration of 2 wt.%; however, a longer aging period of 1 month was required. The increase in activity after one month in the latter case could be explained by surface changes in QA-PEI nanoparticles, which resulted in an increase in the number of active sites.

## 4. Properties of Dental Composite Restorative Materials Modified with QA-PEI Nanoparticles

Quaternary ammonium PEI derivatives, including QA-PEI nanoparticles, are recognized as compounds with selective cytotoxicity towards bacteria and fungi while remaining harmless to mammalian cells [33,86,107,108]. Therefore, QA-PEI nanoparticles, added at low concentrations, have the potential to be effective biocides for dental composite applications. So far, a series of commercial dental composite restorative materials have been modified by the physical admixing of 1 to 2 wt.% QA-PEI nanoparticle powder and tested for antibacterial and physico-mechanical properties (Table 3). For determining the antimicrobial activity of dental materials modified with QA-PEI nanoparticles, Direct Contact Test (DCT), and the Agar Diffusion Test (ADT) are the most common methods. In addition, inhibition zone measurement is typically applied to detect the diffusion of biocide. The biological response is most often tested against *S. mutans* [80,85,92,93], which is the main cause of secondary dental caries [109,110,111]. However, other bacteria strains are also tested in this work (Table 3). In addition to the microbiological tests, the modified dental materials are tested regarding the degree of conversion (DC) in the dimethacrylate composite matrix and their mechanical properties, such as flexural strength and flexural modulus.

The antibacterial tests on the modifications of commercial dental composite materials with QA-PEI nanoparticles were carried out with QA-PEI nanoparticle content from 0.5 to 2.0 wt.%. The presence of 1 wt.% QA-PEI nanoparticles mostly triggered satisfactory antibacterial activity.

After incorporation of 1 wt.% QA-PEI nanoparticles, Filtek Z250 (3M^TM^) and Filtek Flow (3M^TM^) achieved strong and long-lasting (over one month) surface antibacterial activity against *S. mutans* [80,85,89]. The inhibition zone was not observed, which indicates that QA-PEI nanoparticles were not eluted from the composite. The modification of the Filtek Flow composite with QA-PEI nanoparticles caused an increase in the hydrophobic character of its surface, which was manifested by an increase in the water contact angle [89]. Flexural tests on Filtek Z250 hybrid composite did not reveal any relevant effects for the modification with QA-PEI nanoparticles. The flexural strength of the Filtek Flow flowable composite was more sensitive to the addition of QA-PEI nanoparticles. Its value decreased by approximately 40% in comparison to the neat composite, whereas the modulus remained unaffected [80,85].

Filtek Flow (3M^TM^) enriched with 1 and 2 wt.% QA-PEI nanoparticles also showed antibacterial activity against *S. aureus*, *S. epidermis*, *E. faecalis*, *P. aeruginosa*, and *E. coli* [90]. The complete growth inhibition of *S. aureus* and *E. faecalis* was achieved for the composite modification with 1 wt.% QA-PEI nanoparticles. The complete growth inhibition of *S. epidermis*, *P. aeruginosa*, and *E. coli* required a higher content of 2 wt.% QA-PEI nanoparticles. The addition of 1 wt.% QA-PEI nanoparticles only reduced the bacterial growth on the surface of these composites. Generally, this diversification of results coincided with the classification of bacterial species into Gram-positive and Gram-negative bacteria. Composites modified with Gram-negative bacteria, *P. aeruginosa* and *E. coli*, showed weaker biological responses than those modified with the Gram-positive bacteria *S. aureus* and *E. faecalis*. Exceptionally, *S. epidermis*, the Gram-positive strain, was more resistant to the biocidal action of QA-PEI nanoparticles than the other tested Gram-positive bacteria. These results supported the conclusion that the specific structure of the cell wall of the Gram-negative bacteria reduced the antibacterial efficiency of QA-PEI nanoparticles [65,66,99,100,101,105]. An inhibition zone was not observed around all the modified composites, which showed that leaching of QA-PEI nanoparticles did not take place.

The Filtek Supreme XT flowable restorative system (3M^TM^) after modification with QA-PEI nanoparticles was tested for antibacterial activity against a series of Gram-positive bacteria: *E. faecalis* [83,88,93], *S. mutans*, *A. viscosus*, *L. casei*, and whole saliva bacteria [93]. Satisfactory antibacterial activity was achieved for composites modified with 1 wt.% QA-PEI nanoparticles.

The Filtek Supreme XT composite modified with 2 wt.% QA-PEI nanoparticles were subjected to further testing regarding the degree of conversion in the dimethacrylate matrix and antibacterial activity against *E. faecalis* relative to the distribution of nanoparticles throughout the sample mass [93]. X-ray photoelectron spectroscopy revealed the presence of iodide cations on both the outer and inner surfaces of the modified composite. However, the concentration of nanoparticles was higher on the inner surface. Despite this fact, total bacterial growth inhibition was achieved for both tested surfaces. The degree of conversion (DC) in the dimethacrylate matrix was not negatively affected by the addition of 2 wt.% QA-PEI nanoparticles. The difference between the DC values determined for unmodified versus modified composites was statistically insignificant and corresponded to around 51% and 53%, respectively. The neutral effect of the modification of dental resin-based material with QA-PEI nanoparticles is particularly important for its proper functioning. It is well known that mechanical and physico-chemical properties of the methacrylate-based dental materials strongly depend on the DC. A decrease in the DC results in the mechanical weakening of the composite [8,102].

Due to the modification with 1 wt.% QA-PEI nanoparticles, commercial dimethacrylate-based foundation material Q Core (BJM Laboratories) showed antibacterial activity against *A. viscosus* and *S. mutans* [92]. As both *A. viscosus* and *S. mutans* represented the Gram-positive bacterial strain, both bacteria revealed similar antibacterial efficiency. The quantity of bacteria accumulated on the surfaces of the materials modified with QA-PEI nanoparticles decreased by six orders of magnitude relative to unmodified materials.

The influence of polishing on the antibacterial activity of a commercial composite was also tested. Polishing is an integral part of dental filling procedure as it provides optimal aesthetics and marginal integrity of the restorative interface [116]. The test results on the antibacterial activity of the Q Core (BJM Laboratories) commercial dental composite enriched with 1 wt.% QA-PEI nanoparticles against *A. viscosus* and *S. mutans* did not reveal any negative effect of polishing on the biocidal efficiency of the modified material [92].

## 5. QA-PEI Nanoparticles in Other Dental Materials

Dental plaque accumulation and the formation of secondary caries can also be associated with the use of other types of dental materials, such as orthodontic cements, adhesives, and endodontic sealers. Thus, some studies investigated those types of materials after modification by incorporating QA-PEI nanoparticles in order to determine if such modifications could increase their antibacterial activity against oral bacteria (Table 4).

Endodontic sealers are utilized for the obturation of root canals. Studies on their modification to achieve materials of biocidal activity are particularly important for modern dentistry. As the root canal constitutes the ultimate barrier between the oral cavity and inner body, it must be particularly protected against bacterial infections. The modification of the commercial epoxy-amine root canal sealer pastes, including AH Plus, AH26 (Dentsply DeTrey), and BJM RCS (B.J.M. Laboratories) with wt.1% QA-PEI nanoparticles, resulted in total bacterial growth inhibition against *E. faecalis* [88].

Commercial dental cements and adhesives were also tested for modification with QA-PEI nanoparticles. They are used in orthodontics for securing brackets to the teeth and for luting artificial teeth in place. In recent years, there has been considerable growth in the use of adhesive systems to repair teeth damaged by caries [117,118,119].

The Single Bond (3M^TM^) adhesive modified with 1 wt.% QA-PEI nanoparticles showed antibacterial activity against *S. mutans* [80]. Moreover, the inhibition zone was not observed around the sample.

The provisional commercial dental cement, RelyX Temp NE (3M ESPE), was another material that showed antibacterial activity against *S. mutans* and *E. faecalis* due to modification with 1wt.% QA-PEI nanoparticles. No bacterial growth was observed on samples aged up to 14 days, which could be recognized as long-lasting antibacterial activity [97].

The modification of the NeoBond (Dentsply) orthodontic cement with 1.5 wt.% QA-PEI nanoparticles resulted in the complete inhibition of *S. mutans* bacterial growth and did not change material biocompatibility [94]. Another study showed that the same orthodontic cement, enriched with 1 wt.% QA-PEI nanoparticles, reduced and inhibited the bacterial growth of *S. mutans* and *L. casei* in the oral biofilm adjacent to bonded brackets by approximately 90% [120]. Additionally, the bacterial biomass around orthodontic brackets was reduced by more than 40%. Therefore, this method can be recognized as advantageous in preventing the development of caries adjacent to orthodontic bracket devices.

**Figure 12:**
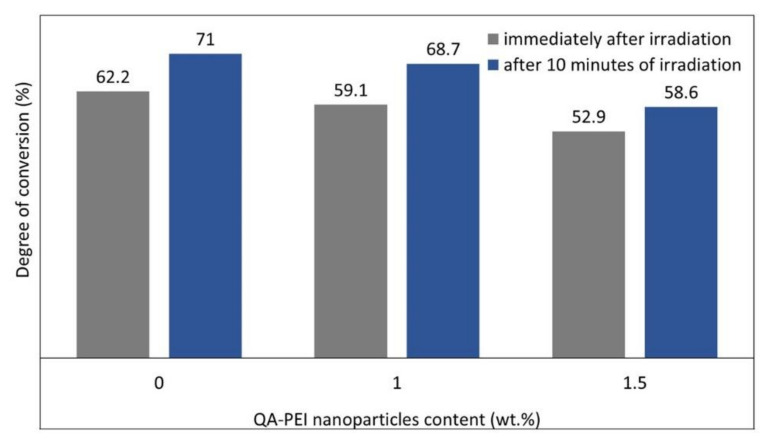
Degree of conversion of orthodontic adhesives modified with QA-PEI nanoparticles. Data from [94].

The study on the NeoBond (Dentsply) orthodontic adhesive revealed that the DC in the methacrylate matrix could decrease due to the addition of QA-PEI nanoparticles (Figure 12). The presence of 1 wt.% QA-PEI nanoparticles caused a 3% decrease in the DC measured 10 min after irradiation, whereas the presence of 1.5 wt.% QA-PEI nanoparticles caused a 17.5% decrease in the DC, relative to the neat material. The influence of the decrease in the DC on the material mechanical performance was tested by measuring the shear bond strength (SBS). The results did not show a negative effect on SBS.

Orthodontic cements and adhesives modified with QA-PEI nanoparticles do not always show sufficient antibacterial activity. Among the studied orthodontic adhesives NeoBond (Dentsply), Transbond Plus (3M Oral Care), Transbond CT (3M Oral Care), and orthodontic cements GC Fuji ORTHO LC (GC), GC CX-Plus (SHOFU), only the NeoBond adhesive and GC Fuji ORTHO LC cement exhibited strong and long-lasting antibacterial activity against *S. mutans* due the modification with 1 wt.% QA-PEI nanoparticles. This effect was observed even after one month [95]. Modification of these two materials with the QA-PEI nanoparticles reduced the bacterial counts by about 95% and 97%, respectively. No inhibition zone was observed around any of the tested samples.

Additionally, we would like to present the results for the modification of commercial bone cement with QA-PEI nanoparticles. Bone cements are based on poly(methyl methacrylate) (PMMA), which represents a linear polymer that is structurally different from the crosslinked dimethacrylate-based dental materials. Therefore, the results achieved for this type of biomaterial can be valuable for PMMA-based dental materials, such as artificial teeth, denture bases, dentures, obturators, orthodontic retainers, temporary or provisional crowns, and for the repair of dental prostheses [121]. Simplex TM P Bone Cement (Stryker MedEd), due to the incorporation of 2 wt.% QA-PEI nanoparticles showed antibacterial activity against *S. aureus* and *E. faecalis* [96]. The inhibition zone was not observed around the tested samples, so it could be concluded that no amount of antibacterial agent was leached from the bone cement structure. Young’s modulus of the modified materials was determined in order to evaluate the influence of QA-PEI nanoparticles on the mechanical properties of the cement. The Young’s modulus values decreased from 2.31 GPa for the neat cement to 1.9 GPa for the cement enriched with 2 wt.% QA-PEI nanoparticles. However, the difference between those values was found to be statistically insignificant. The modulus of the material containing 3 wt.% QA-PEI nanoparticles was 1.8 GPa, which was significantly statistically lower than the modulus value of the unmodified material.

## 6. Biocompatibility of Dental Materials Modified with QA-PEI Nanoparticles

Certain commercial dental materials modified with QA-PEI nanoparticles were tested for biocompatibility and were subjected to initial in vivo testing.

Generally, dental materials in their cured form are non-toxic. However, they can reveal certain toxic effects to the human body due to leaching, which usually results from the presence of unreacted monomers and low molecular weight products of the chemical degradation of the dimethacrylate polymer network [102]. In the case of dental materials modified with QA-PEI nanoparticles, cytotoxicity could result from the presence of QA-PEI nanoparticles and/or their leaching [102]

The cytotoxicity of the QA-PEI modified dental materials was tested using macrophages and fibroblast cell lines and studying its effect on the secretion of tumor necrosis factor alpha (TNFα) from the macrophages.

The addition of 1 wt.% QA-PEI nanoparticles into commercial dental materials did not negatively affect their biocompatibility [89,90,91,98]. This effect was demonstrated on two commercial dental restorative composites, Filtek Z250, and Filtek Flow [89,90,91], as well as three commercial endodontic sealers, AH Plus (Dentsply Maillefer), Epiphany SE (Pentron Clinical Technologies), and GuttaFlow (Coltene/Whaledent) [98]. The cell viability was observed for each of the tested materials on both cell lines.

The increase in the amount of QA-PEI nanoparticles of up to 2 wt.% led to the diversification of results.

The dental restorative composites Filtek Z250 and Filtek Flow, modified with 2 wt.% QA-PEI nanoparticles, did not reveal an increase in the material cytotoxicity. The cytotoxic effect was comparable with that of the native composite [89,90,91]. On that basis, it can be concluded that the addition of a maximum 2 wt.% QA-PEI nanoparticles does not alter the cytotoxicity of restorative dental composite materials. Moreover, it can be concluded that the dimethacrylate component (composite matrix) is the main source of cytotoxic effects in dental composite materials containing QA-PEI nanoparticles.

On the other hand, the incorporation of 2 wt.% QA-PEI nanoparticles into tested endodontic sealers was recognized as slightly toxic [98]. Although the cell viability was observed for each of the tested sealers on both cell lines, the reduction in the TNFα secretion was observed for the modified AH Plus sealer. The modified Epiphany sealer reduced the TNFα secretion to an undetectable level, whereas the modification of GuttaFlow did not affect the TNFα secretion (in comparison to the unmodified sealers). This diversification of results is a very important outcome for the future design of dental materials modified with QA-PEI nanoparticles. The maximum content of QA-PEI in a dental material may vary depending on the intended use of the material, but also on the manufacturer and particular product.

The results for the modification of endodontic sealers with QA-PEI nanoparticles are particularly interesting. As commercial endodontic sealers usually contain substances recognized as cytotoxic, such as eugenol, paraformaldehyde, or polyketone [122], their replacement with QA-PEI nanoparticles seems to be an interesting alternative.

Initial in vivo studies on the QA-PEI-modified dental material were also performed on the Filtek Flow restorative composite. The implantation of the modified restorative, containing up to 2 wt.% QA-PEI nanoparticles, into rat tissue indicated that an inflammatory reaction [89].

Satisfactory results were also achieved for another in vivo test for the Filtek Flow composite modified with 1 wt.% QA-PEI nanoparticles [91]. The modified and non-modified materials were inserted into a removable acrylic appliance. The device was worn by 10 volunteers for four hours to allow the build-up of intraoral biofilms. Then, the specimens were tested for bacterial vitality and biofilm thickness. The results indicated that composites containing QA-PEI nanoparticles exhibited antibacterial activity against salivary bacteria and were safe for oral use because no inflammation nor allergic reactions were sustained. The vitality rate in the QA-PEI nanoparticle-modified composites was reduced by more than 50%, whereas biofilm thickness increased. The thicker biofilm that accumulated on the surfaces of composites containing QA-PEI nanoparticles primarily consisted of nonviable cells, whereas the thinner biofilm that was detected on the surfaces of control samples consisted mainly of viable cells. The in vitro tests showed a 70% decrease in viable bacteria due to the modification of the dental composites with QA-PEI nanoparticles.

## 7. Conclusions and Future Perspectives

The lack of antibacterial properties of commonly-used dental composite materials is the main problem associated with providing restoration longevity and avoiding surrounding tissue irritation. Dental materials can be enriched with various organic and inorganic biocides in order to give them microbiological activity. Many of them are known to have adverse effects on surrounding tissues or on human health in broad terms. Therefore, their leachates trigger a toxic response. QA-PEI nanoparticles represent an interesting alternative. Due to their polycationic character, resulting from the ammonium group in the repeating units, they exhibit strong and long-lasting antibacterial activity. Their nanoparticulate form allows application in small amounts; therefore, their distribution within a dental composite does not deteriorate the material’s overall physico-chemical and mechanical properties. Additionally, these nanoparticles offer a wide range of potential chemical modifications, which results in a specific antibacterial activity. Since they do not increase the cytotoxicity of the modified dental materials, they can be recognized as being safe for mammalian cells.

This review shows that the structure of QA-PEI nanoparticles has to be carefully designed to achieve optimum antibacterial activity. It depends on the particular elements of the chemical structure and bacterial strain and may be summarized as follows: (i) polycationic character provides higher antibacterial activity if compared to monocationic compounds, (ii) both the excessive core stiffness and elasticity decreases antibacterial efficiency, (iii) the optimum dibromopentane crosslinker concentration is around 0.04 mol/mol PEI unit, (iv) *N*-octyl is the optimum *N*-alkyl substituent, (v) the optimum *N*-alkylation ratio is 1 mole/PEI unit, (vi) the quaternization should be full, (vii) the antibacterial activity against Gram-positive bacteria is stronger than that against Gram-negative bacteria, (viii) many bacterial strains show QA-PEI antimicrobial susceptibility, (ix) when added in 1 wt.% into the commercial dental material, satisfactory antibacterial action is usually achieved.

Further systematic studies on the QA-PEI structure-bioactivity relationships are of great interest. They can lead to the development of materials presenting an optimal balance between antimicrobial activity and cytotoxicity. Moreover, extending research to other microorganisms can bring new potential applications. The examination of antiviral properties is particularly interesting from the perspective of the SARS-CoV-2 (COVID-19) pandemic.

The modification of dental materials with QA-PEI nanoparticles brings the following outcomes: (i) they are physically admixed, which represents the cheapest and easiest method of material physico-chemical modification; (ii) good antibacterial activity is achieved at 1 wt.% content; (iii) this content does not cause an increase in the original material cytotoxicity; (iv) 2 wt.% content causes a slight increase in the material cytotoxicity; (v) 1 wt.% content does not display harmful effects on living tissues; (vi) the nanoparticulate crosslinked form provides non-leaching properties; (vii) their presence does not decrease the degree of conversion in the composite; (viii) their presence does not cause deterioration in its flexural properties.

In view of the above, it can be concluded that the modification of dental materials with QA-PEI nanoparticles presents a viable alternative to currently used dental biocides. Nevertheless, QA-PEI modified materials have not yet been fully characterized and require further investigation. In particular, the following properties need to be examined: (i) mechanical behavior under tensile and compressive loading; (ii) other mechanical properties, such as hardness and abrasion resistance; (iii) glass temperature; (iv) water sorption; (v) polymerization shrinkage; (vi) degradation effects caused by the exposure to UV, pH (in respect to particular bacterium strains), and water (via Hofmann elimination); (vii) the biological and physical effects of long-lasting leaching. As the biocompatibility has only been initially assessed, it also requires further examination and in vitro as well as in vivo tests.

Although QA-PEI nanoparticles were originally designed to provide dental materials with antibacterial activity against oral bacteria, they may also prove to be suitable for other biomedical applications. Therefore, further studies on the modifications of other composite biomaterials with QA-PEI nanoparticles are highly desirable.

## Figures and Tables

**Table 1:** The influence of the crosslinker type on the antimicrobial activity of quaternary ammonium polyethylenimine (QA-PEI) nanoparticles against *S. aureus* and *P. aeruginosa*. Data from [84].

Alkyl Bromide.	Crosslinking Agent	Antibacterial Activity ^1^	
		*S. aureus*	*P. aeruginosa*
1-bromohexadecane	Ethylene glycol bis(chloroacetate)	3.2	<1.5
	Diethylene glycol bis(chloroacetate)	3.4	<1.5
	Triethylene glycol bis(chloroacetate)	3.6	<1.6
	Polyethylene glycol bis(chloroacetate)	3.8	<1.7
	1,5-Dibromopentane	2.1	<1.5
	Glutaraldehyde	2.4	<1.5

^1^ Antibacterial activity was calculated using Equation (1).

**Table 2:** Antibacterial activity of QA-PEI nanoparticles prepared with the ethylene glycol bis(chloroacetate) crosslinker and different alkylation agent chain lengths against *S. aureus* and *P. aeruginosa*. Data from [84].

Alkyl Bromide	Crosslinking Agent	Crosslinking Ratio	Antibacterial Activity ^1^	
			*S. aureus*	*P. aeruginosa*
1-bromooctane	Ethylene glycol bis(chloroacetate)	1:0.02	>6	5.0
		1:0.04	>6	4.3
		1:0.06	>6	3.8
		1:0.08	4.8	2.5
		1:0.1	4.6	<1.8
1-bromododecane	Ethylene glycol bis(chloroacetate)	1:0.02	4.9	1.6
		1:0.04	4.4	<1.5
		1:0.06	4.2	<1.5
		1:0.08	3.7	<1.5
		1:0.1	3.3	<1.5
1-bromohexadecane	Ethylene glycol bis(chloroacetate)	1:0.02	3.5	<1.5
		1:0.04	3.2	<1.5
		1:0.06	2.8	<1.5
		1:0.08	2.6	<1.5
		1:0.1	1.8	<1.5

^1^ Antibacterial activity was calculated using Equation (1).

**Table 3:** Commercially-available dental composite materials tested for antibacterial activity after modification with QA-PEI nanoparticles.

Dental Composite Material/Manufacturer	Chemical Composition	Content of QA-PEI Nanoparticles (wt.%)	Tested Bacteria	Ref.
Filtek^TM^ Z250/3M^TM^	Zirconia/silica filler, Bis-GMA, UDMA, Bis-EMA [112]	1	*S. mutans*	[80]
Filtek Flow/3M^TM^	Zirconia/silica filler, Bis-GMA, TEGDMA	1	*S. mutans*	[80,85]
		1 2	*S. aureus*, *S. epidermis*, *E. faecalis*, *P. aeruginosa*, *E. coli*	[90]
		1	*in vivo* studies	[91]
Single Bond/3M^TM^	Dimethacrylate resins, HEMA [113]	1	*S. mutans*	[80]
Q Core/BJM Laboratories Ltd.	Bis-GMA, TEGDMA, aluminoborosilicate filler, fluoride-releasing filler	1	*A. viscosus*, *S. mutans*	[92]
Filtek Supreme XT Flowable Restorative/3M^TM^	Zirconia/silica filler Bis-GMA, TEGDMA, Bis-EMA [114]	0.5 1 2	*E. faecalis*	[83,88]
Filtek Supreme XTE Flowable Restorative/3M^TM^	Zirconia/silica filler, fluoride filler, Bis-GMA, TEGDMA, procrylat resins [115]	1 2	*E. faecalis*, *S. mutans*, *A. viscosus*, *L. casei*	[93]

**Table 4:** Commercially-available dental materials tested for antibacterial activity after modification with QA-PEI nanoparticles.

Dental Composite Material/Manufacturer	Type of Dental Material	Content of QA-PEI Nanoparticles (wt.%)	Tested Bacteria	Ref.
AH Plus/Dentsply DeTrey	Root canal sealer pastes	0.5 1 2	*E. faecalis*	[88]
AH 26/Dentsply DeTrey	Root canal sealer pastes	0.5 1 2	*E. faecalis*	[88]
BJM RCS/B.J.M. Laboratories	Root canal sealer pastes	0.5 1 2	*E. faecalis*	[88]
Single Bond adhesive/3M^TM^	Bonding resin	1	*S. mutans*	[80]
RelyX Temp NE/3M ESPE	Provisional cement	0.5 1 2	*S. mutans* *E. faecalis*	[97]
NeoBond	Orthodontic cement	1 1.5	*S. mutans*	[94]
		1	*S. mutans* *L. casei*	[117]
		1	*S. mutans*	[95]
Transbond Plus/3M Oral Care	Orthodontic adhesive	1	*S. mutans*	[95]
Transbond CT/3M Oral Care	Orthodontic adhesive	1	*S. mutans*	[95]
GC Fuji ORTHO LC/GC	Orthodontic cement	1	*S. mutans*	[95]
GC CX-Plus/SHOFU	Orthodontic cement	1	*S. mutans*	[95]
Simplex TM P Bone Cement/Stryker MedEd	Bone cement	1 2 3	*S. aureus* *E. faecalis*	[96]
